# Onco-testicular sperm extraction (onco-TESE) for bilateral testicular tumors: two case reports

**DOI:** 10.1186/s13256-017-1303-6

**Published:** 2017-05-17

**Authors:** Sohgo Tsutsumi, Takashi Kawahara, Teppei Takeshima, Sawako Chiba, Koichi Uemura, Masako Otani, Kota Shimokihara, Yutaro Hayashi, Taku Mochizuki, Daiji Takamoto, Yusuke Hattori, Jun-ichi Teranishi, Yasuhide Miyoshi, Masahiro Yao, Yoshiaki Inayama, Yasushi Yumura, Hiroji Uemura

**Affiliations:** 1 Department of Urology/Renal Transplantation, Yokohama City University Medical Center, Yokohama, Japan; 2 Reproduction Center, Department of Urology, Yokohama City University Medical Center, Yokohama, Japan; 3 Division of Diagnostic Pathology, Yokohama City University Medical Center, Yokohama, Japan; 4 Department of Urology, Hiratsuka Kyosai Hospital, Hiratsuka, Japan; 5 Department of Urology, Yokohama City University Graduate School of Medicine, Yokohama, Japan

**Keywords:** Onco-TESE, Azoospermia, Oligospermia, Testicular cancer

## Abstract

**Background:**

Most patients with testicular cancer are infertile; thus, the preservation of the sperm after surgery is an important factor to consider. We report two cases of bilateral testicular cancer in patients who underwent bilateral higher orchiectomy and simultaneous testicular sperm extraction.

**Case presentation:**

Two Asian-Japanese men were referred to our hospital with bilateral testicular tumors. Both of the patients were preoperatively diagnosed with azoospermia and requested testicular sperm extraction at the time of higher orchiectomy. In one patient, sperm was successfully harvested and then frozen. In the other patient, sperm could not be retrieved from the patient’s testis. In both patients, the pathological diagnosis was seminoma. Testicular tumors often occur in patients of reproductive age. The preservation of sperm before chemotherapy or bilateral orchiectomy is necessary for patients with testicular tumors who wish to be fathers.

**Conclusions:**

Onco-testicular sperm extraction might be an option for patients with testicular cancer and azoospermia or severe oligospermia.

## Background

The standard treatment for testicular tumors is higher orchiectomy. Adjuvant chemotherapy and radiation therapy are sometimes added. Most patients with testicular cancer are infertile; thus, preservation of their sperm after surgery is an important factor to consider. We report two cases of bilateral testicular cancer in patients who underwent bilateral higher orchiectomy and simultaneous testicular sperm extraction (onco-TESE). Sperm was successfully harvested from one of these patients.

## Case presentations

### Patient 1

**Fig. 1:**
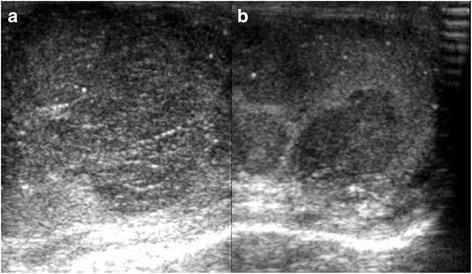
Ultrasonographic findings in patient 1. **a** Right testis. **b** Left testis

**Fig. 2:**
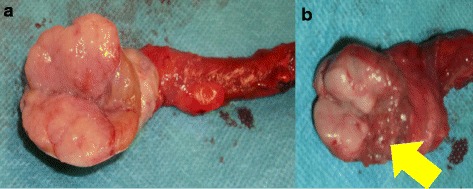
Macroscopic findings in patient 1. **a** Right testis. **b** Left testis (*Arrow*: normal testis)

**Fig. 3:**
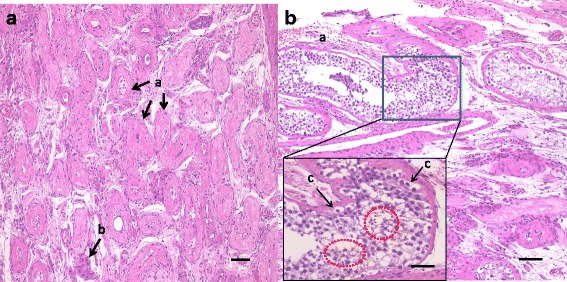
Histopathological findings using hematoxylin and eosin stain. **a** Shrunken seminiferous tubules (*a*) and Leydig cells (*b*) (bar = 100 μm). **b** Seminiferous tubules (*a*) (bar = 100 μm); *inset*: spermatogonia (*c*); *red circles*: spermatids (bar =50 μm) Mean Johnsen’s score count: **a** 1.40, **b** 4.38. *Arrow*: normal tetis

A 38-year-old unmarried Asian-Japanese man with bilateral testicular tumors was referred to our hospital to undergo sperm preservation. His right testis was swollen to the size of a chicken’s egg; his left testis was normally sized but had a nodule. He had no particular medical history. A laboratory examination showed almost normal results (including lactate dehydrogenase [LDH] and α-fetoprotein [AFP]), with the exception of the human chorionic gonadotropin (HCG) level, which was elevated (25.8 mIU/ml). The patient’s serum testosterone level was within the normal range. An ultrasonographic examination showed a low echoic lesion on the whole right testis and partially on the left testis at the same location as the palpable nodule (Fig. 1a and b). Computed tomography (CT) showed no distant metastasis or lymph node swelling. On the basis of these findings, bilateral testicular tumors (cTxN0M0) were diagnosed, and bilateral orchiectomy was planned. In a sperm examination performed 1 day before surgery, no sperm were detected in 8.2 ml of semen. Owing to the patient’s azoospermia, we planned to perform onco-TESE from his normal left testicular tumor at the time of bilateral orchiectomy. Because the tumorous lesion was easily detected macroscopically, we could retrieve the sperm of his normal left testicular tumor soon after resection. Approximately 1000 sperm were counted and then frozen. The pathological diagnosis was seminoma (pT1N0M0). Most of the right testis was occupied by the tumor. Microscopically, the testicular parenchyma adjacent to the tumor was atrophic, with shrunken seminiferous tubules having thickened basement membranes and diminished numbers of germ cells, a so-called Sertoli cell-only appearance or completely hyalinized. Collections of hyperplastic Leydig cells were also seen. The patient’s mean Johnsen score was 1.40 (Figs. 2a and 3a). In the left testis, the parenchyma adjacent to the tumor was also atrophic and showed an almost Sertoli cell-only appearance, but it contained a few seminiferous tubules with sperm (mean Johnsen score 4.38) (Figs. 2b and 3b). The patient’s HCG level was normal after surgery, and he has received testosterone supplementation since surgery. He did not receive adjuvant chemotherapy and has had no recurrence in the 0.5 years since surgery.

### Patient 2

**Fig. 4:**
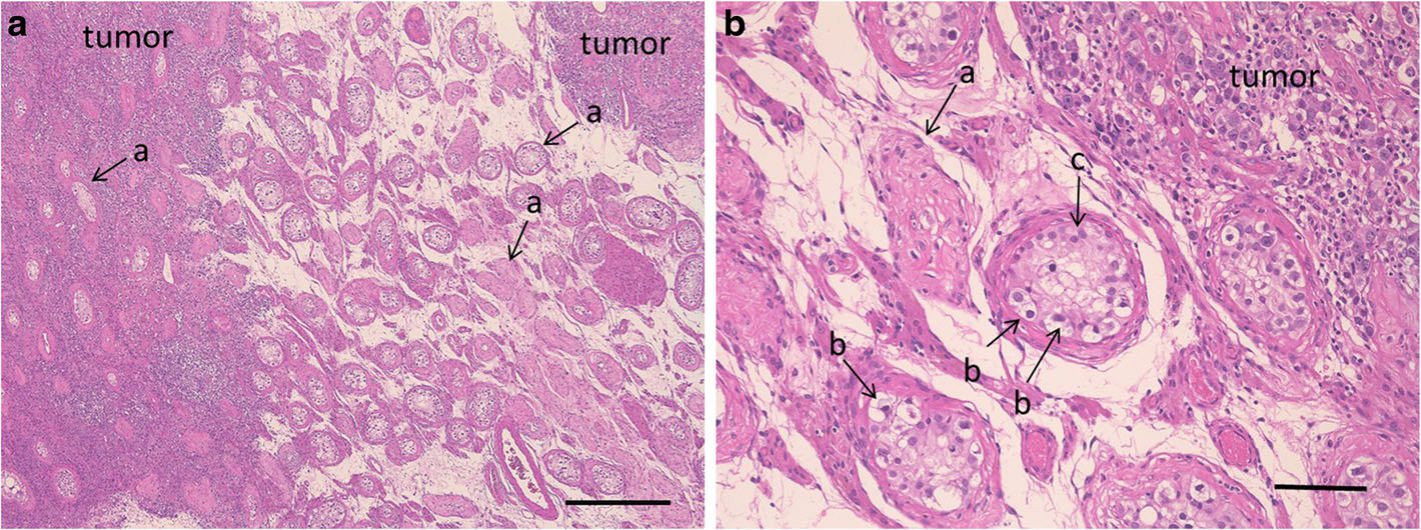
Histopathological findings using hematoxylin and eosin stain. **a** Seminiferous tubules (bar = 500 μm). **b** Spermatogonia (bar = 100 μm). **c** Sertoli cells

A 35-year-old married Asian-Japanese man was referred to our hospital with bilateral swelling of the scrotum. He had no particular medical history. A blood analysis revealed elevated tumor marker levels (LDH 314 IU/L, HCG 27.4 mIU/ml). The patient’s AFP level and other laboratory data were within the normal limits. CT showed no distant or lymph node metastasis. On the basis of these findings, the patient was preoperatively diagnosed with a bilateral testicular tumor (cTxN0M0). One day prior to bilateral orchiectomy, a semen test was performed, but no sperm were identified (semen volume 1.8 ml). We planned to perform onco-TESE at the time of surgery because the patient wanted to preserve his sperm. Grossly, a white multinodular tumor occupied the patient’s entire left testis, whereas the tumor of the right testis left a compressed area of uninvolved parenchyma (Fig. 4). After the resection of both testes, we examined the normal part of the right testis microscopically, but no sperm were detected. The pathological diagnosis was seminoma (pT1N0M0). Microscopically, the uninvolved parenchyma of the right testis showed atrophy. In some areas without gross abnormality, tumor cells invaded between preserved seminiferous tubules. Spermatocytes were noted in preserved seminiferous tubules, but spermatogenesis was arrested. The patient’s mean Johnsen score was 2.40. The patient’s elevated LDH and HCG levels decreased to a normal level after surgery, and no adjuvant therapy was administered. He received testosterone supplementation after surgery and has shown no recurrence in the 2.5 years since surgery.

## Discussion

Testicular tumors account for 1.0–1.5% of malignant disease in male patients, and they usually occur in men 15–35 years of age. Bilateral cases account for 1.0–2.0% of testicular tumors. Heterochronous bilateral testicular tumors are seen four times more frequently than simultaneous bilateral testicular tumors. Seminoma, which is the most common type of bilateral testicular tumor, is seen in 47% of heterochronous cases and 67% of simultaneous cases [1]. Although bilateral cases require testosterone replacement therapy, the prognosis is almost the same as that in unilateral cases.

The risk of testicular tumors in male infertility patients is 20 times higher than that in patients who are not infertile. The higher incidence of testicular tumors in infertile patients was thought to be associated with the diminished spermatogenic function of the patients before the development of the tumor. This hypothesis is referred to as *testicular dysgenesis syndrome*. The syndrome is suggested to be a reason for the development of testicular tumors, a decrease in spermatogenic function, cryptorchidism, and hypospadia [2]. For these reasons, male infertility patients should be screened for testicular tumors.

Onco-TESE is a procedure that allows sperm to be obtained from the normal testis of patients who do not emit sperm prior to cancer therapy. Onco-TESE can be applied to patients with azoospermia, severe oligospermia, or ejaculation disorder before chemotherapy or in cases involving bilateral testicular tumors. Sperm was frozen for a total of 168 cases at our institute from 2011 to 2016. Fifty-three (31.5%) of these cases involved patients with testicular tumors. Twenty-six (60.5%) of these cases involved patients with bilateral testicular tumors (bilateral, *n* = 6; unilateral, *n* = 20). Five of these patients were azoospermic, and one patient was dysspermic. We performed onco-TESE in three patients. Including our own patients, 43 cases of onco-TESE have been reported in the literature [3–6]. In 22 (51.2%) cases, sperm was successfully preserved, including 14 patients (53.8%) with testicular tumors. The previous reports noted that pregnancy was achieved in four cases using sperm extracted via onco-TESE, with a healthy child delivered in three of the four cases. A correlation between tumor size and spermatogenesis in patients with testicular tumors has been reported. Jeremy *et al*. reported that spermatogenesis was observed in 86%, 81%, and 57% of cases involving tumors of 1 cm, 2 cm, and 5 cm in size, respectively [7].

Although onco-TESE is useful, the number of institutes in which surgeons perform the procedure is limited. When these restrictions are cleared, onco-TESE will become an important option for preserving fertility. Regarding infertility, CT might have some effects. But, based on cancer control, CT examination should be recommended.

## Conclusions

We describe two cases in which onco-TESE and resection were performed simultaneously in patients with bilateral testicular tumors.
